# Excessive supraventricular ectopic activity and new-onset ischemic stroke: a retrospective cohort study with prospective outcome ascertainment

**DOI:** 10.3389/fneur.2026.1921826

**Published:** 2026-09-29

**Authors:** Min Yang, Yujie Tang, Haiyan Jiang, Chaochen Yin, Ruijuan Gang, Zhaoxia Ran, Zepeng Lai, Pengyuan Wang, Yuetong Luo, Rong Wang, Qi Wang, Xiong Yu, Qiushuo Liu, Chenlin Hu, Mingtian Zhuang, Qi Lin, Qi Wu, Yapeng Lin

**Affiliations:** 1 Department of Neurology, Zigong Fourth People’s Hospital, Zigong, China; 2 School of Clinical Medicine, Chengdu Medical College, Chengdu, China; 3 The First Affiliated Hospital of Chengdu Medical College, Chengdu, China; 4 Department of Medical Affairs Office, The People’s Hospital of Jianyang City, Chengdu, China; 5 School of Public Health, Chengdu Medical College, Chengdu, China; 6 Department of Cardiology, Chengdu Seventh People’s Hospital (Affiliated Cancer Hospital of Chengdu Medical College), Chengdu, China; 7 Department of Cardiology, The Second Affiliated Hospital of Chengdu Medical College, Nuclear Industry 416 Hospital, Chengdu, Sichuan, China; 8 Aging Mechanisms and Interventions Key Laboratory of Sichuan Province, Chengdu Medical College, Chengdu, China; 9 Key Laboratory of Intelligent Medical Care and Elderly Health Management, Chengdu, China

**Keywords:** electrocardiogram, excessive supraventricular ectopic activity, mortality, paroxysmal atrial tachycardia, premature atrial complex, stroke

## Abstract

**Background:**

Excessive supraventricular ectopic activity (ESVEA), a marker of both paroxysmal atrial fibrillation (pAF) and underlying atrial cardiomyopathy, may serve as an earlier electrographic predictor of cryptogenic ischemic stroke than pAF itself. This study aimed to investigate the association between ESVEA and the risks of new-onset ischemic stroke and all-cause mortality in an Asian population, and to explore a potential data-driven threshold that requires external validation.

**Methods:**

In this retrospective cohort study with prospective outcome ascertainment, data were collected from 446 patients without a history of atrial fibrillation or stroke across three hospitals in China. Based on the results of 24-h ambulatory ECG monitoring, patients in the top 15% of the cohort (premature atrial complex [PAC] rate ≥100 beats/24 h or paroxysmal atrial tachycardia [PAT] episode count ≥22 episodes/24 h) were defined as the ESVEA group; the remainder comprised the non-ESVEA group. The incidence of new-onset ischemic stroke and all-cause mortality was compared between groups during follow-up, after adjustment for confounding factors such as age and hypertension.

**Results:**

The median follow-up duration was 13 months. In a multivariable Cox proportional hazards model with a time-varying coefficient for age, using new-onset ischemic stroke and all-cause mortality as endpoints, after adjusting for other risk factors, ESVEA was significantly associated with new-onset ischemic stroke (HR:2.798; 95% CI:1.064 to 7.359; *p* = 0.037) and all-cause mortality (HR:7.584; 95% CI: 1.302 to 44.163; *p* = 0.024). In subgroup analysis, no significant interaction was observed between age groups for the effect of ESVEA on new-onset ischemic stroke. The rates of new-onset stroke in the ESVEA- group (without PAC/PAT), the ESVEA+/− group (with PAC/PAT not meeting ESVEA criteria), and the ESVEA+ group were 0.70, 5.90, and 16.30%, respectively, demonstrating a stepwise increase.

**Conclusion:**

In this retrospective cohort study with prospective outcome ascertainment, ESVEA was associated with an increased risk of new-onset ischemic stroke and all-cause mortality in Asian subjects without prior stroke or AF. These exploratory findings suggest that ESVEA may be a useful risk marker, but require validation in larger prospective studies before clinical application.

## 1 Introduction

Approximately 25%–30% of ischemic strokes are cryptogenic ischemic strokes and pAF is considered one of the presumed etiologies (1–4). However, long-term rhythm monitoring detects occult pAF in only a small subset of patients. Thus, in recent years, it has been proposed that ESVEA, as a manifestation or marker of occult pAF and underlying atrial myopathy, may serve as an earlier predictive indicator for cryptogenic ischemic stroke risk than atrial fibrillation itself, thereby holding promise as an alternative key marker for risk stratification of new-onset ischemic stroke (5–7).

The association between ESVEA and new-onset ischemic stroke has been increasingly studied, yet a clear consensus has not been established (8–12). Furthermore, most of these studies have focused on the European population, and their applicability to the Asian population remains unclear. This gap is clinically relevant because stroke subtype profiles and cardiovascular risk profiles differ between Asian and Western populations, for example, a relatively higher proportion of intracranial large-artery atherosclerosis and small-vessel disease has been reported in Asian populations, and the burden and clinical significance of PAC/PAT may also differ across ethnic groups. Another key issue is that the clinical threshold of ESVEA for predicting stroke and other adverse outcomes has not been standardized (13, 14). For example, >500 PACs/24 h (15), ≥30 PACs/h or any episode of runs of ≥20 PACs (11), PACs >100 beats/day (16), >97 PACs/h (17), etc. Compared with PAC burden alone, PAT episodes represent longer, more organized runs of supraventricular activity and may carry distinct thromboembolic implications; because PAT burden has received less emphasis in previous predominantly Western cohorts, it was included as a co-defining component of ESVEA in this study. Therefore, further research is urgently needed to explore the association between ESVEA and new-onset ischemic stroke, as well as the exploratory ESVEA threshold that may be associated with adverse outcomes—especially in the Asian population.

This study aims to explore the association between ESVEA and new-onset ischemic stroke, to propose exploratory thresholds for ESVEA that may be associated with new-onset ischemic stroke and other adverse outcomes in an Asian population, and thereby to generate hypotheses that may inform future primary prevention strategies for ischemic stroke.

## 2 Methods

### 2.1 Study design and population

This was a multicenter, retrospective, observational cohort study conducted at three large general hospitals in China, covering the period from July 2023 to November 2024. The baseline characteristics, 24-h ambulatory ECG data, and medication records were collected retrospectively from the electronic medical records, whereas outcome ascertainment during follow-up was carried out prospectively through standardized telephone interviews every 6 months. The study adhered to the ethical principles of the Declaration of Helsinki and received approval from the Institutional Review Board of The First Affiliated Hospital of Chengdu Medical College (approval number: 2025CYFYIRB-SQ-28), the Ethics Committee of Nuclear Industry 416 Hospital (approval number: YJ-2024-004-02), and the Ethics Committee of Chengdu Seventh People’s Hospital (approval number: QT2021-084-03). Written informed consent was obtained from all participating patients.

Inclusion criteria: (1) Age 18–80 years; (2) Patients who did not meet the WHO criteria for stroke, with no evidence of hemorrhagic or ischemic stroke on brain computed tomography (CT) or magnetic resonance imaging (MRI); (3) Availability of 24-h ambulatory ECG diagnostic results; (4) Signed informed consent from the patient or family members. It should be noted that the clinical indications for 24-h ambulatory ECG monitoring in this study, as documented in medical records, included evaluation of palpitations, dizziness or syncope, arrhythmia screening in patients with hypertension or diabetes, and preoperative cardiovascular risk assessment. Exclusion Criteria: (1) History of previous stroke; (2) Diagnosis of atrial fibrillation (AF) or atrial flutter (AFL) on 24-h ambulatory ECG; (3) Patients with severe cardiac, pulmonary, hepatic, or renal dysfunction; (4)Inability to comply with the study requirements.

The final analysis involved 446 patients. Based on previous literature (16) and assuming that PAC and PAT need to be excessive to significantly elevate the risk of adverse events, patients with a supraventricular arrhythmia burden in the top 15% of the cohort (PAC rate ≥100 beats/24 h or PAT episode count ≥22 episodes/24 h) were assigned to the ESVEA group, and the remaining patients were allocated to the non-ESVEA group. Given the lack of a standardized ESVEA definition for Asian populations, this exploratory, cohort-distribution-based threshold was adopted on the basis of previous literature and the clinical rationale that PAC and PAT burdens must be excessive to increase the risk of adverse events. Additionally, patients were further divided into three groups during subgroup analysis: (1) ESVEA- group: without PAC/PAT; (2) ESVEA+/− group: with PAC/PAT not meeting ESVEA criteria; (3) ESVEA+ group: PAC rate ≥100 beats/24 h or PAT episode count ≥22 episodes/24 h. In this three-category classification, “without PAC/PAT” (the ESVEA- group) denotes patients in whom neither premature atrial complexes nor paroxysmal atrial tachycardia were detected on the 24-h ambulatory ECG, whereas the ESVEA+/− group comprises patients in whom PACs and/or PAT episodes were present but did not reach the ESVEA threshold.

### 2.2 Baseline examinations

Baseline demographic data, potential confounding factors (hypertension, diabetes mellitus, hyperlipidemias, coronary disease, smoking, myocardial infarction, heart failure), results of 24-h Holter monitoring, and medication use were collected from electronic medical records using a standardized data collection form. The CHA₂DS₂-VASc score (Congestive heart failure, Hypertension, Age ≥ 75 years, Diabetes mellitus, Previous stroke or transient ischemic attack [TIA], Vascular disease, Age 65–74 years, Sex category [female]) was calculated (18). All 24-h ambulatory electrocardiograms were retrospectively collected and reviewed by the researchers. ESVEA was quantified by the 24-h PAC rate (PACs/24 h) and PAT episode count (PAT episodes/24 h).

### 2.3 Endpoint determination and follow-up

The primary outcome of this study was new-onset ischemic stroke, and the secondary outcome was all-cause mortality. Outcome events were collected through standardized telephone interviews by trained personnel during blinded follow-up. The interviews were performed every 6 months. Outcome events were verified and supplemented by reviewing patients’ electronic medical records and results of 24-h ambulatory ECG re-examinations. Repeat 24-h ambulatory ECG was performed only when clinically indicated, such as new-onset palpitations or syncope, and not systematically for all patients. Data from all three centers were analyzed centrally. The diagnosis of new-onset ischemic stroke was based on clinical manifestations and confirmed by brain CT or MRI; only cases with imaging confirmation were included, with all images independently reviewed by two blinded investigators using the WHO diagnostic criteria. Any disagreements were resolved by a third investigator. The follow-up duration was defined as the number of months from the date of enrollment (baseline assessment) until the occurrence of new-onset ischemic stroke, all-cause mortality, loss to follow-up, or the end of follow-up. In addition, during follow-up, researchers re-inquired about patients’ medication use from discharge to follow-up based on the medication information collected at baseline to obtain more accurate data.

### 2.4 Statistical analysis

Continuous variables are presented as mean ± standard deviation (SD) if normally distributed; otherwise, they are expressed as median (interquartile range). Categorical variables are summarized as frequency (percentage). Intergroup comparisons were conducted using the Mann–Whitney U test for continuous and ordinal variables, and the Pearson chi-square test, continuity-corrected chi-square test, or Fisher exact test for categorical variables, as appropriate.

The Cox proportional hazards model was used to assess the hazard ratios (HRs) for the associations between ESVEA and the endpoints of brain infarction or all-cause death. The selection of potentially confounding covariates was based on existing knowledge of the relationship between ESVEA and brain infarction. Furthermore, variables with statistically significant differences between groups at baseline or factors associated with cerebral infarction in univariate Cox regression analysis were also included based on the criterion of *p* < 0.1. However, to avoid multicollinearity, we did not include the CHA₂DS₂-VASc score. The final model included a pre-specified set of clinically established covariates (age, sex, hypertension, diabetes mellitus, hyperlipidemia, smoking, and use of anticoagulants or antiplatelet agents); univariate Cox regression served only as an auxiliary screening check. The proportional hazards assumption for the Cox models was assessed graphically using Schoenfeld residual plots, log–log plots of survival time, and cumulative hazard function plots. Given the presence of a time-varying coefficient for age, we adjusted the Cox model using the time-dependent coefficient method, which was implemented by adding an age*time interaction term. Furthermore, we conducted subgroup analyses for two predefined subgroups using Cox proportional hazards models and tested for interaction effects by including interaction terms. Kaplan–Meier survival function and the log-rank test were used to compare the survival probabilities for new-onset ischemic stroke and all-cause mortality. A two-sided *p*-value < 0.05 was considered statistically significant. All statistical analyses were performed using SPSS version 26.0.

## 3 Results

### 3.1 Study population

This study enrolled a total of 446 patients, with a median age of 59.00 years, and 51.8% were female. Based on 24-h ambulatory ECG monitoring, 43 patients were classified as the ESVEA group. The median follow-up duration was 13 months. A total of 10 patients were lost to follow-up (overall loss-to-follow-up rate: 2.24%), all in the non-ESVEA group, and were excluded from the outcome analysis. Loss to follow-up was mainly due to the inability to contact patients. The baseline characteristics of the two groups are presented in Table 1. Among the baseline variables of interest, only age, CHA₂DS₂-VASc score, and the use of anticoagulants showed a significant association with ESVEA. Of the 446 included patients, four were receiving oral anticoagulant therapy at baseline. Indications included calf muscular venous thromboembolism (*n* = 2), post-ablation thromboprophylaxis for myopathic atrial tachycardia (*n* = 1), and postoperative thromboprophylaxis after knee arthroplasty (*n* = 1). None of the patients received anticoagulation for atrial fibrillation or for primary or secondary stroke prevention, as atrial fibrillation was an exclusion criterion.

**Table 1:** baseline characteristics of the subjects with and without ESVEA.

Variable	All	ESVEA group (*N* = 43)	Non-ESVEA group (*N* = 403)	*p*-value
Age, y, median (IQR)	59.0 (50.0–70.0)	68.0 (59.0–73.0)	59.0 (50.0–69.0)	0.001
Sex, no. (%)				
Male	215 (48.2)	23 (53.5)	192 (47.6)	0.466
Female	231 (51.8)	20 (46.5)	211 (52.4)	
CHA2DS2-VASc score, no. (%)				
0	66 (14.8)	2 (4.7)	64 (15.9)	0.015
1	150 (33.6)	13 (30.2)	137 (34.0)	
2	112 (25.1)	11 (25.6)	101 (25.1)	
≥3	118 (26.5)	17 (39.5)	101 (25.1)	
Relevant medical history, no. (%)				
Heart failure	9 (2.0)	0 (0.0)	9 (2.2)	1.000
Myocardial infarction	2 (0.4)	0 (0.0)	2 (0.5)	1.000
Hypertension	160 (35.9)	15 (34.9)	145 (36.0)	0.887
Diabetes mellitus	70 (15.7)	11 (25.6)	59 (14.6)	0.061
Hyperlipidemias	96 (21.5)	9 (20.9)	87 (21.6)	0.921
Coronary disease	22 (4.9)	2 (4.7)	20 (5.0)	1.000
Smoking	79 (17.7)	12 (27.9)	67 (16.6)	0.065
Medications, no. (%)				
Anti-arrhythmia agents	25 (5.6)	5 (11.6)	20 (5.0)	0.145
Anticoagulants	4 (0.9)	2 (4.7)	2 (0.5)	0.048
Antiplatelet agents	26 (5.8)	2 (4.7)	24 (6.0)	0.996
Antihypertensive agents	85 (19.1)	9 (20.9)	76 (18.9)	0.742
Hypoglycemic agents	40 (9.0)	4 (9.3)	36 (8.9)	1.000
Hypolipidemic agents	48 (10.8)	4 (9.3)	44 (10.9)	0.947
Follow-up(month), median (IQR)	13.0 (12.0–16.0)	13.0 (12.0–16.0)	13.0 (12.0–17.0)	0.239

The patient cohort was stratified according to the presence of ESVEA. The *p*-value of the CHA2DS2-VASc score is a test of the overall within-group difference. ESVEA = excessive supraventricular ectopic activity; IQR = interquartile range; CHA2DS2-VASc = congestive heart failure, hypertension, age ≥75 years, diabetes mellitus, previous stroke or TIA, vascular disease, age 65–74 years, sex category [female]. Follow-up duration is not a baseline characteristic but is reported in Table 1 to allow comparison of follow-up completeness and censoring between the groups.

### 3.2 Primary and secondary outcomes

During the follow-up period, 7 of the 43 patients (16.3%) in the ESVEA group were diagnosed with new-onset ischemic stroke, and 4 (9.3%) died. In contrast, among the 393 patients in the non-ESVEA group, 16 (4.1%) experienced new-onset ischemic stroke and 5 (1.3%) died (Table 2). Cause-of-death data were obtained through telephone follow-up with family members for all 9 patients who died during the follow-up period. The reported causes were predominantly malignancy (*n* = 5) or cardiopulmonary disease (*n* = 2), with the remaining two deaths attributed to severe pneumonia (*n* = 1) and post-fracture malnutrition with infection (*n* = 1). None of the 9 deaths was directly attributable to ischemic stroke. In the initial multivariable Cox model adjusted for baseline risk factors, ESVEA was significantly associated with new-onset ischemic stroke (HR: 2.740; 95% CI: 1.042 to 7.211; *p* = 0.041) and all-cause mortality (HR: 7.681; 95% CI: 1.319 to 44.721; *p* = 0.023). Since the variable age violated the proportional hazards assumption, we further constructed a final model incorporating a time-varying coefficient for age (Table 3). After adjusting for age and sex, ESVEA remained significantly associated with both new-onset ischemic stroke (HR: 3.272; 95% CI: 1.331 to 8.045; *p* = 0.010) and all-cause mortality (HR: 5.528; 95% CI: 1.458 to 20.962; *p* = 0.012). These associations persisted after further adjustment for additional risk factors (hypertension, diabetes mellitus, hyperlipidemia, smoking, and the use of anticoagulants or antiplatelet agents), with ESVEA remaining significantly associated with both new-onset ischemic stroke (HR: 2.798; 95% CI: 1.064 to 7.359; *p* = 0.037) and all-cause mortality (HR: 7.584; 95% CI: 1.302 to 44.163; *p* = 0.024). Consistently, the Kaplan–Meier curves showed that patients with ESVEA had a significantly higher risk of new-onset ischemic stroke (log-rank *p* = 0.001) and all-cause mortality (log-rank *p* < 0.001) than those in the non-ESVEA group (Figure 1).

**Table 2:** Primary and secondary outcomes.

Outcome	ESVEA group (*N* = 43)	Non-ESVEA group (*N* = 393)	Adjusted HR (95% CI)
Primary outcome			
New-onset ischemic stroke. no. (%)	7 (16.3)	16 (4.1)	2.798 (1.064–7.359)
Secondary outcome			
All-cause mortality. no. (%)	4 (9.3%)	5 (1.3)	7.584 (1.302–44.163)

HR = hazard ratio; CI = confidence interval. HR (95% CI) are from the final Cox model (Table 3). ESVEA = excessive supraventricular ectopic activity.

**Table 3:** Cox regression models showing the HR of ESVEA in relation to new-onset ischemic stroke and all-cause mortality.

Model	Without a time-varying coefficient for age		With a time-varying coefficient for age (final model)	
	New-onset ischemic stroke	All-cause mortality	New-onset ischemic stroke	All-cause mortality
ESVEA in Model 1^*^	3.189 (1.297–7.845)	5.567 (1.470–21.087)	3.272 (1.331–8.045)	5.528 (1.458–20.962)
	*p* = 0.012	*p* = 0.012	*p* = 0.010	*p* = 0.012
ESVEA in Model 2^†^	2.740 (1.042–7.211)	7.681 (1.319–44.721)	2.798 (1.064–7.359)	7.584 (1.302–44.163)
	*p* = 0.041	*p* = 0.023	*p* = 0.037	*p* = 0.024

Values are hazard ratios (95% confidence interval).

*Model 1: adjusted for age and sex.

^†^Model 2: adjusted for age, sex, hypertension, diabetes mellitus, hyperlipidemias, smoking, anticoagulants, and antiplatelet agents. ESVEA = excessive supraventricular ectopic activity.

**Figure 1:**
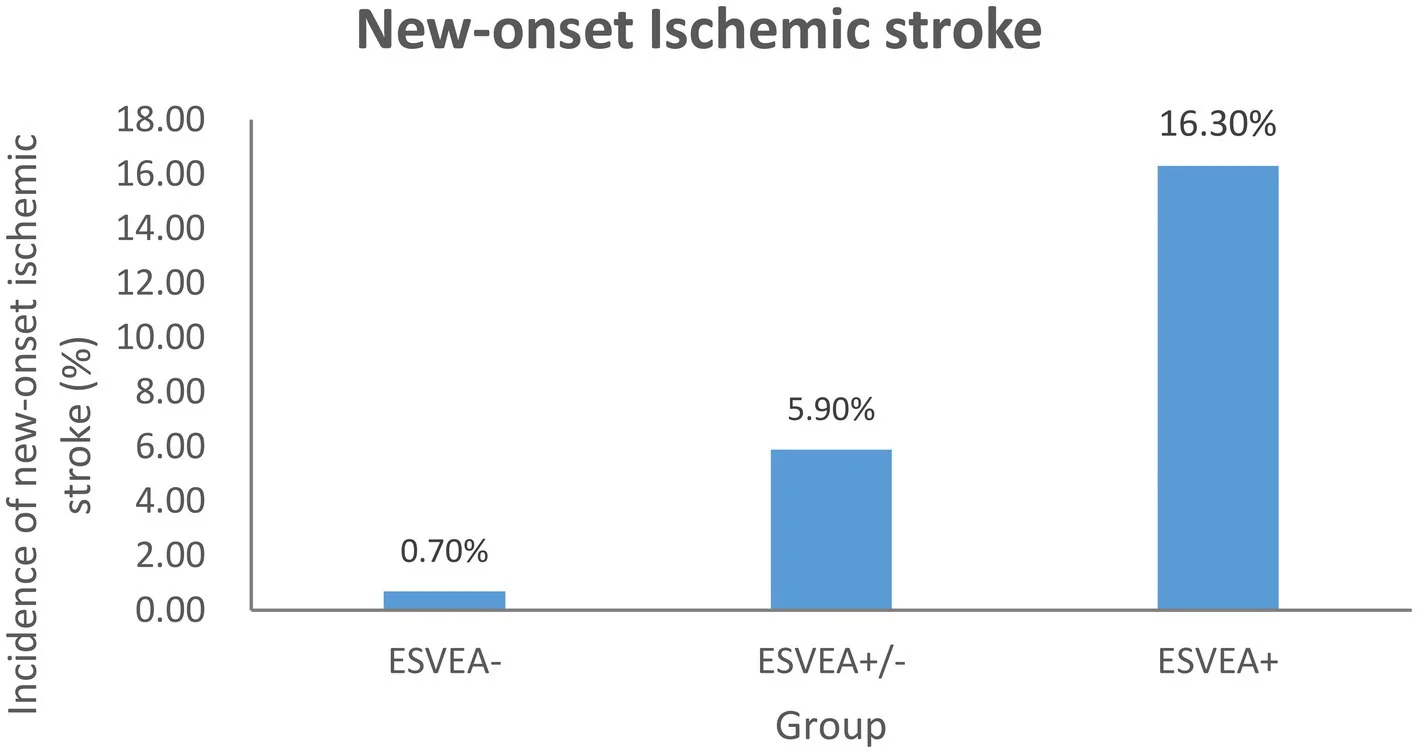
New-onset ischemic stroke rates in ESVEA−, ESVEA+/−, and ESVEA+. Data are presented as percentages, showing a stepwise increase with increasing supraventricular arrhythmia load.

### 3.3 Subgroup analysis

The results of the subgroup analyses by age and by the three-category grouping (ESVEA+, ESVEA+/−, and ESVEA-) are presented in Table 4. Patients were stratified into two age groups: ≤65 years (*n* = 275) and >65 years (*n* = 161). Within the ≤65 years and >65 years subgroups, the incidence of new-onset ischemic stroke among ESVEA patients was 11.8% (2/17) and 19.2% (5/26), respectively. The test for interaction revealed no significant effect modification by age on the association between ESVEA and new-onset ischemic stroke (P for interaction = 0.372). Furthermore, patients in the non-ESVEA group were subdivided into the ESVEA+/− group and ESVEA− group based on the presence of PAC or PAT. The incidence of new-onset stroke in the ESVEA− group, ESVEA+/− group, and ESVEA+ group was 0.70, 5.90, and 16.30%, respectively, showing a stepwise increase (Figure 2). Pairwise comparisons with Bonferroni correction demonstrated a statistically significant difference between the ESVEA+ group and the ESVEA− (HR: 15.621; 95% CI: 1.813 to 134.607; *p* = 0.012). The ESVEA+/− group showed a trend toward increased risk relative to the ESVEA− group that did not reach statistical significance (HR: 7.702; 95% CI: 0.994 to 59.669; *p* = 0.051). No significant differences were observed between the ESVEA+ group and the ESVEA+/− group (HR: 0.493; 95% CI: 0.184 to 1.319; *p* = 0.159).

**Table 4:** Subgroup analysis of the primary outcome.

Group	Presence (+) or absence (−) of ESVEA	N	New-onset stroke, no.(%)^*^	*p*-value	HR (95% CI)
Age					
≤65	+ESVEA	17	2 (11.8)	0.146	3.956 (0.620–25.241)
	−ESVEA	258	4 (1.6)		
>65	+ESVEA	26	5 (19.2)	0.266	1.988 (0.593–6.669)
	−ESVEA	135	12 (8.9)		
P for interaction	0.372				
Three categories					
ESVEA+ vs. ESVEA−				0.012	15.621 (1.813–134.607)
ESVEA+/− vs. ESVEA−				0.051	7.702 (0.994–59.669)
ESVEA+ vs. ESVEA+/−				0.159	0.493 (0.184–1.319)

HR = hazard ratio; CI = confidence interval. HR (95% CI) are from the final Cox model (Table 3). *Indicates % of number in the row. ESVEA = excessive supraventricular ectopic activity; ESVEA+ = PAC rate ≥100 beats/24 h or PAT episode count ≥22 episodes/24 h; ESVEA+/− = with PAC/PAT not meeting ESVEA criteria; ESVEA− = without PAC/PAT.

**Figure 2:**
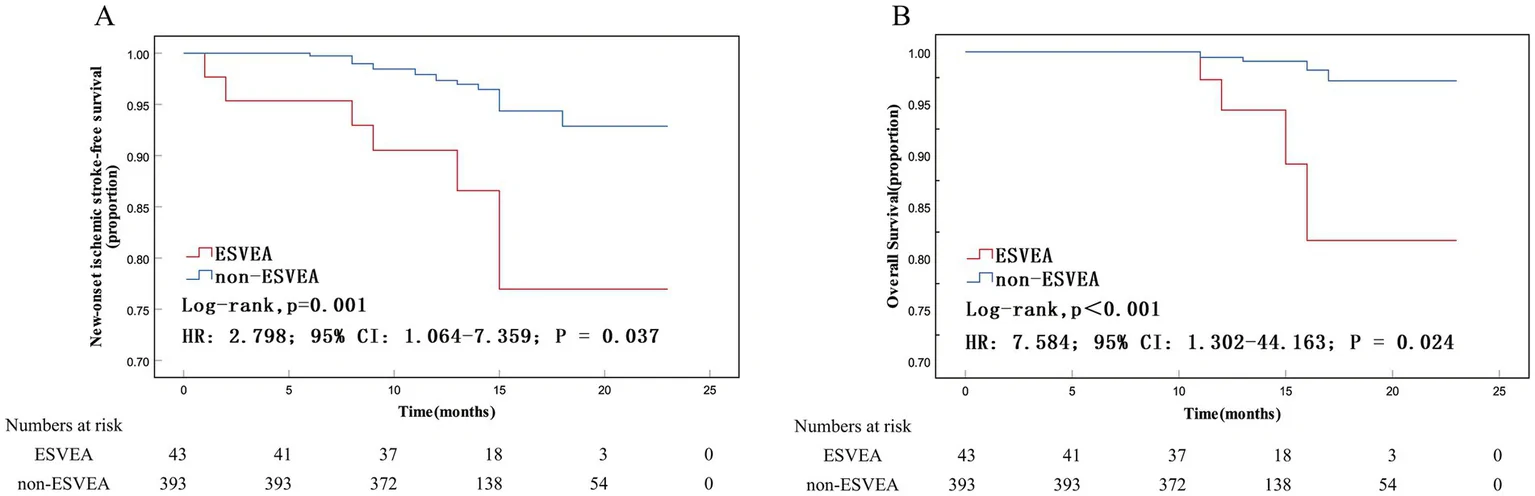
Kaplan–Meier estimate for new-onset ischemic stroke-free and overall survival, stratified by ESVEA. Kaplan–Meier survival estimate of new-onset ischemic stroke-free survival **(A)** and overall survival **(B)** in patients with and without ESVEA.

## 4 Discussion

### 4.1 Summary of main findings

Our study demonstrated that ESVEA was significantly associated with new-onset ischemic stroke and all-cause mortality in the Asian population. No significant interaction was observed between age groups for the effect of ESVEA on new-onset ischemic stroke. The incidence rate of new-onset stroke showed a stepwise increase among the ESVEA- group, the ESVEA+/− group and the ESVEA+ group.

### 4.2 Comparison with existing literature and heterogeneity findings

At present, AF and AFL have been established as definite risk factors for new-onset ischemic stroke, necessitating anticoagulation therapy for prevention (19–21). However, in recent years, a growing body of research has focused on the association between supraventricular arrhythmias other than AF and AFL and ischemic stroke (13, 14). The findings of this study indicate that ESVEA is significantly associated with new-onset ischemic stroke and all-cause mortality, which is consistent with the results of numerous previous studies. For instance, Engström et al. demonstrated that a high frequency of atrial ectopic beats (AEB) is associated with an increased incidence of stroke (22). Kamel et al. (23) study found an independent association between paroxysmal supraventricular tachycardia (PSVT) and ischemic stroke. Our findings can be contextualized by comparison with the landmark Copenhagen Holter Study. Binici et al. (10). reported 6.3-year stroke rates of 10% in the ESVEA group and 3% in the non-ESVEA group in a healthy Caucasian population. In our Asian hospitalized cohort, the 13-month stroke rates were 16.3% in the ESVEA+ group and 4.1% in the non-ESVEA group. Because the follow-up durations differed substantially (6.3 years vs. 13 months), these raw event proportions are not directly comparable. Notably, the relative risk estimate for ESVEA was similar in the two studies (HR 2.79 in the Copenhagen study vs. HR 2.80 in the fully adjusted model of our study), suggesting that the association between ESVEA and stroke is consistent across populations and follow-up durations. The differences in absolute event rates between the two studies may be attributable to differences in ESVEA definition, comorbidity burden, ethnicity, and sample size. These findings suggest that ESVEA warrants further investigation as a risk marker in Asian populations. The observed stroke rate in the ESVEA group (16.3%) is higher than the threshold typically used to recommend oral anticoagulation in AF patients. Nevertheless, direct evidence supporting DOAC therapy in ESVEA patients without documented AF is lacking. Our findings are hypothesis-generating and should not be interpreted as a call for off-label anticoagulation.

Notably, several previous studies have indicated a close association between ESVEA and new-onset AF; however, no AF events were detected during the entire follow-up period of our study. This may be explained by two factors. First, the median follow-up was only 13 months, which is relatively short for assessing long-term thromboembolic risk. Second, no systematic long-term rhythm monitoring (e.g., implantable loop recorder) was performed; therefore, undetected paroxysmal AF may have occurred and could either mediate or confound the observed association between ESVEA and stroke. Future studies with prolonged cardiac monitoring are needed to clarify the independent role of ESVEA. However, even in the absence of detected AF, patients with ESVEA in our study still exhibited a higher risk of incident ischemic stroke. This finding is consistent with the Copenhagen Holter Study, which reported that 82% of patients with ESVEA did not develop clinical AF during a 15-year follow-up period (11), suggesting that ESVEA may be an indicator that precedes atrial fibrillation and is associated with new-onset ischemic stroke and other adverse outcomes.

To date, no consensus has been reached regarding the threshold of ESVEA for predicting ischemic stroke or other adverse outcomes (13). In studies utilizing ambulatory electrocardiogram monitoring, the definition of ESVEA is highly heterogeneous, such as >500 PACs/24 h (15), ≥30 PACs/h or any episode of runs of ≥20 PACs (11), PACs >100 beats/day (16), >97 PACs/h (17), etc. This study defines ESVEA as ≥100PACs/24 h or ≥22 PAT episodes/24 h, providing evidence for the future standardization of the ESVEA definition. Furthermore, there is a paucity of previous studies on this topic focusing on the Asian population. A Japanese study demonstrated that supraventricular premature complexes (SVPCs) were associated with an increased risk of stroke-related mortality in patients without documented AF, but failed to clarify their association with ischemic stroke (24). Additionally, a case–control study in Chinese Taiwan indicated that PSVT was a significant risk factor for ischemic stroke (25). Building on this foundation, the present study further supplements evidence regarding the association between ESVEA and ischemic stroke in the Asian population.

### 4.3 Potential mechanisms

Several mechanisms have been proposed to explain how ESVEA contributes to ischemic stroke. One hypothesis is that ESVEA increases the risk of ischemic stroke and all-cause mortality by identifying patients who are at risk of developing AF (12, 26). Another hypothesis is that ESVEA may serve as a marker of atrial cardiomyopathy, contributing to increased thrombosis and embolism by inducing left atrial dilation, left atrial appendage stasis, and myocardial fibrosis (27, 28). Additional studies have also proposed that ESVEA is associated with multiple risk factors for stroke, such as hypertension and diabetes mellitus, thereby increasing the risk of ischemic stroke (6, 8). In the present study, it was also found that the average age of patients in the ESVEA group was significantly higher than that in the non-ESVEA group, and they were more likely to suffer from hypertension, coronary artery disease, and other diseases predisposing to arrhythmias-overlapping with the traditional risk factors for ischemic stroke. However, after adjusting for confounding factors, ESVEA remained significantly associated with incident ischemic stroke, which may reflect residual confounding from unmeasured or imprecisely measured factors, such as left atrial structural abnormalities or inflammatory markers. Furthermore, Huang et al. emphasized that PACs can serve as a sign of non-cardiac diseases or a marker of subclinical cardiovascular disease, which provides an additional reference for explaining the association between ESVEA and ischemic stroke (29). Although we proposed several mechanisms for how ESVEA contributes to ischemic stroke, findings from a large-scale multicenter randomized controlled trial (RCT) failed to demonstrate the benefit of oral anticoagulant therapy in an unselected population with cryptogenic ischemic stroke (30). This suggests the heterogeneity of mechanisms underlying cryptogenic ischemic stroke, and further research is needed to improve the identification of patients with cryptogenic ischemic stroke of cardiac origin in the future. Notably, none of the deaths in this study was directly attributable to ischemic stroke. Consequently, the observed association between ESVEA and all-cause mortality likely reflects underlying comorbidities rather than a direct causal effect of ESVEA on fatal stroke. Given the limited number of events, this finding should be considered exploratory and hypothesis-generating.

### 4.4 Study strengths

Compared with previous approaches that prevent embolic events by identifying high-risk patients with occult AF, using ESVEA as a predictive marker is more feasible, as ESVEA occurs more frequently than AF episodes. The exploratory findings of our study for Asian populations include: (1) we propose an exploratory, cohort-specific threshold (top 15% of PAC/PAT burden) that may serve as a hypothesis-generating reference for future validation in Asians; (2) we show a stepwise increase in stroke risk across three categories, including a distinct intermediate-risk group; (3) we report that PAT episode count (≥22/24 h) as an ESVEA component is associated with stroke, which has received less emphasis in previous predominantly Caucasian cohorts.

### 4.5 Study limitations

The primary limitations of this study are its retrospective design and relatively small sample size, which may restrict the statistical power and generalizability of the findings. Additionally, the shorter follow-up of this study compared to previous studies precludes assessment of long-term outcomes. Selection bias is another concern, as all patients underwent 24-h ambulatory ECG monitoring for clinical indications (e.g., palpitations, dizziness, syncope, or arrhythmia screening in high-risk patients) rather than as part of a community-based screening program. Consequently, this cohort may not fully represent the general asymptomatic population, which could limit the generalizability of our findings and influence the direction of effect estimates. Regarding rhythm monitoring, the lack of continuous long-term recording means we cannot rule out the possibility that undetected paroxysmal atrial fibrillation mediates the association between ESVEA and stroke; moreover, some AF or stroke cases may have gone unrecognized during follow-up. In addition, the subtypes of new-onset ischemic stroke (for example, embolic versus non-embolic or cryptogenic) were not systematically adjudicated in the present study, and a formal etiological classification was therefore not available; future studies should classify stroke subtypes in order to clarify the mechanisms that link ESVEA to ischemic stroke. Another important limitation is that the ESVEA threshold (PAC ≥ 100/24 h or PAT ≥22 episodes/24 h) was derived from the same dataset used for outcome analysis, which may introduce optimism bias. Finally, the small number of events resulted in wide confidence intervals and unstable estimates in the subgroup analyses. Therefore, our ESVEA definition and all subgroup findings should be considered exploratory and hypothesis-generating. Moreover, ischemic stroke and all-cause mortality are competing risks. Cause-specific Cox models were used, and a Fine-Gray subdistribution hazard model was not feasible: with 23 ischemic stroke events and at least eight covariates, the events-per-variable ratio would be approximately 2.9, far below the minimum of 10 generally recommended for reliable estimation of the subdistribution hazard. These statistical limitations further underscore the need for validation in larger cohorts. External validation in prospective, independent cohorts (ideally from multiple centers and different ethnic backgrounds) is required before any threshold can be adopted in clinical practice. Future studies with larger-scale prospective designs and extended follow-up are needed to validate our findings.

## 5 Conclusion

In conclusion, our findings further support an association between ESVEA and incident new-onset ischemic stroke and all-cause mortality in an Asian population. We suggest that a comprehensive assessment incorporating multiple risk factors may be considered for primary prevention in patients with ESVEA, pending further validation. Given the exploratory nature of our findings, external validation in larger prospective cohorts is needed before clinical application. Future efforts may include developing a risk prediction model for ischemic stroke in patients with supraventricular arrhythmias other than AF or AFL.

## Data Availability

The raw data supporting the conclusions of this article will be made available by the authors, without undue reservation.
